# Targeting ABC transporters in PDAC – past, present, or future?

**DOI:** 10.18632/oncotarget.28597

**Published:** 2024-06-20

**Authors:** Cecilia Bergonzini, Elisa Giovannetti, Erik H.J. Danen

**Keywords:** PDAC, chemoresistance, ABCB1

Despite its lower incidence as compared to more common cancers such as lung or breast carcinomas, pancreatic ductal carcinoma (PDAC) ranks as the third-leading cause of cancer mortality in the US and the sixth worldwide [1, 2]. This is due to the fact that PDAC survival rates are among the lowest for cancer patients, around 13% in the US [2]. Currently, patients receiving the diagnosis of PDAC are categorized into 4 main groups: resectable, borderline resectable (BRPC), locally advanced (LAPC), or metastatic (non-resectable) [3]. Resectable patients can get the only potentially curative treatment available for PDAC: surgery. Neoadjuvant (pre-operative) chemotherapy can be used to shrink the tumor, potentially eradicate micro-metastases, and increase the chances of candidacy for surgery in BRPC and LAPC patients [4, 5]. Adjuvant (post-operative) chemotherapy can also be applied to reduce the chance of post-operative recurrence of the disease. However, most patients are not resectable at diagnosis and the gold-standard for these patients is chemotherapy using FOLFIRINOX (5-fluorouracil (5-FU), irinotecan, leucovorin, oxaliplatin) or gemcitabine combined with nab-paclitaxel (GnP) [6].

**Figure 1:**
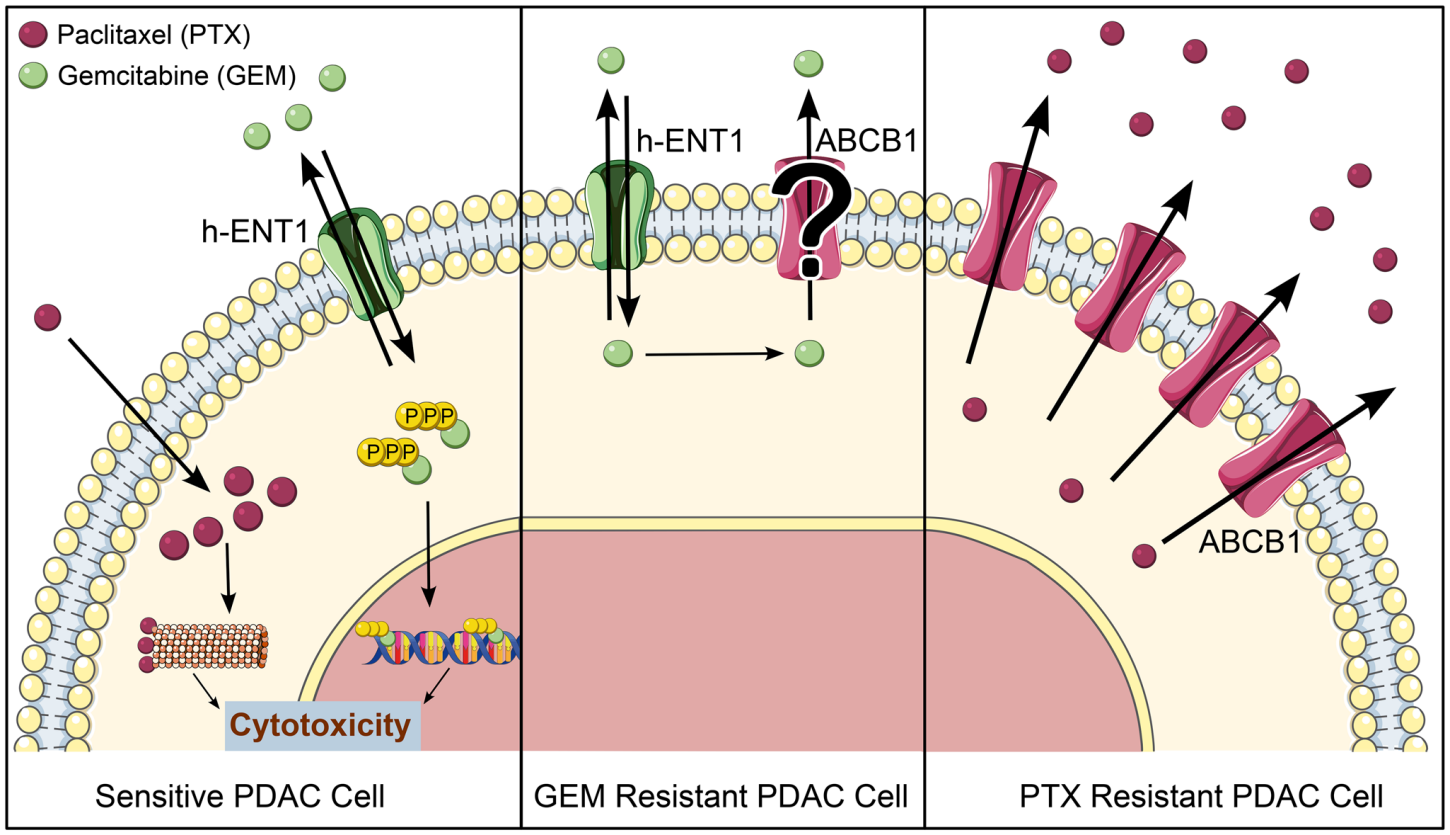
Role for ABCB1 in PDAC chemoresistance. Left, in GnP treated PDAC, paclitaxel and gemcitabine enter PDAC cells and interfere with DNA replication, ultimately causing cytotoxicity. Paclitaxel acts by interfering with microtubule dynamics and phosphorylated gemcitabine acts through incorporation in DNA. Middle, further research is required to reconcile contrasting published data on the role of ABCB1 in the export of gemcitabine from the cytosol as a mechanism of resistance. Right, ABCB1 overexpression drives paclitaxel resistance in PDAC cells, extruding the drug before it can produce a cytotoxic effect.

Gemcitabine (difluorodeoxycytidine, dFdC) is a nucleoside analogue that, upon entering the cell through nucleoside transporters (primarily the human equilibrative nucleoside transporter h-ENT1), undergoes three phosphorylations to become active [7] (Figure 1). The triphosphate form (dFdCTP) is incorporated in DNA and RNA, masked from repair enzymes once a subsequent nucleotide is added, and further DNA/RNA synthesis is arrested [7, 8]. Eventually, this chain of events leads to impaired replication and cell death [9, 10]. Paclitaxel is a microtubule stabilizer that binds β-tubulin heterodimers. It promotes microtubule assembly and inhibits disassembly, which causes arrested mitosis and ultimately cell death [11, 12]. Despite being one of the most common treatments GnP is far from effective. For patients with metastatic disease, it is used to slow the progression, with a median overall survival of 10 months [13]. Inherent or acquired chemoresistance is responsible for a poor prognosis and there is a lack of therapeutic options to overcome it [14]. In fact, the only current alternative in case of progression after GnP is the alternation with FOLFIRINOX or modified 5-FU-based regimens such as FOLFOX or FOLFIRI [6], provided that the health status of the patient allows such treatment adjustments.

ATP-binding cassette (ABC) transporters represent a family of transmembrane proteins that, using the energy from ATP hydrolysis, extrude molecules from the cytoplasm to the exterior or into vesicles [15, 16]. Some of these transporters have been associated with resistance to a spectrum of structurally diverse chemotherapeutic drugs, earning them the name of multidrug resistance (MDR) pumps [17]. One of the best characterized ABC-transporters is ABCB1 (MDR1). It is physiologically expressed in tissues such as kidney, liver, pancreas, intestine, the blood brain barrier, and more, where it exerts a protective role, by extruding xenobiotics and potentially toxic molecules. Moreover, increased ABCB1 expression in tumors has been associated with poor prognosis [16].

Paclitaxel is a bona fide substrate for ABCB1 [18] and ABCB1 has been implicated in paclitaxel and nab-paclitaxel resistance in multiple types of cancer [19, 20]. Could ABCB1 represent a therapeutic target in PDAC patients to suppress resistance against GnP? We have recently reported that ABCB1 can indeed play a critical role in paclitaxel resistance in PDAC cells [21]. We created several PDAC cell models that were made resistant to paclitaxel by exposure to gradually increasing doses. Transcriptomics and proteomics analysis followed by functional validation studies demonstrated that overexpression of ABCB1 was the mechanism driving paclitaxel resistance in all models. This ATP-dependent pump extruded paclitaxel from the resistant PDAC cells and treatment with the chemical ABCB1/ABCC1 inhibitor verapamil or siRNA-mediated silencing of the *ABCB1* gene, re-sensitized the resistant models to paclitaxel. Of note, a recently published abstract reports similar findings using PDAC patient-derived paclitaxel resistant cells [22].

Whether ABCB1 is also involved in gemcitabine resistance is controversial. Several studies suggest a role for ABCB1 in gemcitabine resistance [23–28]. Two of these provide compelling evidence that inhibition of ABCB1 using diltiazem or siRNA counteracts gemcitabine resistance [25, 28]. Interestingly, there are indications based on molecular docking that gemcitabine or dFdCTP could in fact be a substrate of ABCB1 [23, 26]. However, in our paclitaxel resistant models that overexpress ABCB1, no cross-resistance to gemcitabine was observed [21]. Moreover, we developed gemcitabine resistant models using the same strategy as applied for paclitaxel and in these models ABCB1 was not overexpressed, nor did verapamil enhance gemcitabine sensitivity. This argues against a role for ABCB1 in gemcitabine resistance and is in line with earlier reports [29]. Likewise, other ABC transporters have been investigated in the context of gemcitabine resistance with contrasting results [7]. The verdict for the role of ABC transporters in gemcitabine resistance is not out and careful interpretation of the preclinical experiments is required to warrant clinical follow up.

Three generations of ABCB1 inhibitors are currently available [30, 31]. First generation inhibitors, including verapamil, tamoxifen and cyclosporine A lacked sufficient specificity and were too toxic. Second generation inhibitors such as valspodar and dexverapamil were more specific but showed inhibition of cytochrome P450, which necessitated decreased dosing to avoid toxic effects of the chemotherapeutics administered in combination, causing loss of efficacy. The third generation of ABCB1 inhibitors, including tariquidar, zosuquidar, and elacridar have an optimized selectivity and efficacy [32], with tariquidar being able to completely reverse ABCB1 mediated doxorubicin resistance in a breast cancer mouse model [33]. Nevertheless, clinical trials combining ABCB1 inhibitors with chemotherapeutics mostly failed, because of toxicities or a lack of benefits compared to treatment with the chemotherapeutics alone [17, 34, 35].

So, could ABCB1 represent an actionable target in PDAC patients? Our own work showed that ABCB1 is expressed in PDAC patients and detected a trend towards increased overall survival with lower ABCB1 expression in a small cohort of patients receiving GnP as first-line therapy [21]. Other studies confirm expression of ABCB1 in PDAC, with contrasting results on the correlation between expression and survival, which may be related to the selection of patients with respect to the clinical treatment [36, 37]. This demonstrates the importance of screening PDAC patients for expression of ABCB1 to design trials and tailor the therapy to those patients who may benefit from ABCB1 inhibition. Our work also identified a selection of kinase inhibitors that were found to cross react with ABCB1 and sensitize PDAC cells to paclitaxel, thus representing new avenues for combination therapies [21]. Other opportunities in this direction involve advanced drug delivery strategies, to more efficiently and/or selectively inhibit ABCB1 in the target tissue, hence improving the local action of chemotherapy [38, 39]. Lastly, there is a need to advance PDAC models for preclinical studies. Specifically, PDAC cell models that have acquired resistance to the combination of gemcitabine and paclitaxel are lacking but would offer a more realistic representation of the resistance encountered in the patient. On the other hand, PDAC patient-derived organoid libraries are now growing [40–42] providing the opportunity to incorporate inter-patient variability in preclinical studies and test personalized treatment options.

In conclusion, much is still to be learned about this well-known resistance mechanism. This includes a firm understanding of the chemotherapeutics that are- and are not extruded by ABCB1, as well as developing advanced tools for its inhibition in (pancreatic) cancer patients. Moreover, it is crucial for the future application of ABC transporter inhibitors in the clinical setting to develop a stratification protocol, for instance based on ABCB1 overexpression, to identify those PDAC patients who are most likely to benefit from chemosensitization induced by these inhibitors.
